# The murine appendiceal microbiome is altered in spontaneous colitis and its pathological progression

**DOI:** 10.1186/1757-4749-6-25

**Published:** 2014-06-21

**Authors:** Sultan Alkadhi, Dale Kunde, Rajkumar Cheluvappa, Sarron Randall-Demllo, Rajaraman Eri

**Affiliations:** 1 Mucosal Biology Laboratory, School of School of Health Sciences, University of Tasmania, Launceston, TAS, Australia; 2 Department of Medicine, St. George Clinical School, University of New South Wales, Sydney, NSW, Australia

**Keywords:** Microbiome, Appendix, Stool, *Winnie*, Inflammatory bowel disease, Colitis, *Proteobacteria*, *Bacteroidetes*

## Abstract

**Background:**

Inflammatory bowel disease (comprising ulcerative colitis and Crohn’s disease) is a multifactorial disease that is extensively associated with stool microbiome changes (dysbiosis). Appendicitis and appendectomy limits subsequent colitis, clinically, and in animal models. We wanted to examine how the appendiceal and stool microbiome fared in our spontaneous colitic *Winnie* (Muc2^−/−^) mice model.

**Methods:**

Two C57BL/6 and 10 *Winnie* mice at ages 12 and 15 weeks were euthanized for stool and caecal patch samples. DNA was extracted using the QIAamp DNA Stool Mini Kit then the V1-V3 hypervariable region of the 16S rRNA gene was sequenced using the Roche/454 GS FLX + pyrosequencing instrument. A Galaxy metagenomic pipeline was used to define phyla and families at sequence similarity threshold of ≥ 80%.

**Results:**

*Bacteriodetes* was decreased in 15-week *Winnie* mice appendices compared to corresponding stool samples (P < 0.01). *Proteobacteria* was increased in appendices of *Winnie* mice compared to corresponding stool samples (P < 0.05). The *Bacteroidetes* family *Rikenellaceae* could be identified only in 15-week-old *Winnie* mice appendices. A higher quantity of *Acetobacteraceae* (*Proteobacteria* phylum) was present in 15-week *Winnie* mice when compared to 12-week Winnie mice (P < 0.01). *Helicobacteraceae* (*Proteobacteria* phylum), which is prominent in all *Winnie* mice, is absent in control mice.

**Conclusions:**

The appendiceal dysbiosis observed in our *Winnie* mice is commensurate with, and adds to extant literature data. The presence of *Helicobacteraceae* (*Proteobacteria*) only in colitic *Winnie* mice (but not control mice) is consistent with reports of increased *Helicobacter* in IBD patients. *Bacteroides* (*Bacteroidetes*) decreases may be a reflection of reduced anti-inflammatory commensal species such as *B. fragilis*. Further research is warranted to expand and delineate the relationship between IBD and the appendix microbiome.

## Background

Inflammatory bowel disease (IBD) comprises ulcerative colitis (UC) and Crohn’s disease (CD). It has a relapsing and remitting clinical course, and is characterized by chronic intestinal inflammation with abdominal pain and intestinal dysfunction. UC and CD differ in extent, localization, and inflammatory mediator profiles [1]. The etiology of IBD is unknown, but is thought to result from complex interactions between host and environmental components like diet, standard of living, and antibiotic use [2]. IBD is on the rise in developed countries, the latest incidence in Australia being 29.6 per 100,000 [3]. IBD poses a significant economic and public health burden [4].

Gastrointestinal flora (intestinal microbiome) is crucial for human health, mediating important functions in metabolism and immunity [5]. There is a glut of evidence that link the intestinal microbiome to the pathogenesis of IBD [6]. The human microbiome is most concentrated in the colon (10^12^ cells per gram), which is the region most affected in IBD [7]. Manifold studies link IBD with antigen-sensing and intestinal innate immunity genes such as NOD2 and ATG16L1 [8]. Importantly, compositional changes in the intestinal micriobiota (dysbiosis) are significant features of bowel diseases [9]. The correlation between dysbiosis and IBD is not completely understood, and human studies involve confounding variables on bacterial composition, such as antibiotic use [10]. In contrast, animal models of IBD enable the study of the gut microbiome while reducing environmental influence [11]. The *Winnie* mouse strain has missense mutations in the mucin *Muc2* gene that alters the intestinal barrier, and results in “spontaneous” colitis, characterized by intestinal inflammation and activation of the IL-23/Th17 pathway [12]. *Winnie* mice are more vulnerable to intestinal antigens due to defects in Paneth and goblet cells [13], and present with chronologically progressive IBD symptoms such as bloody stools, diarrhoea, and weight loss [12]. All *Winnie* mice (100%) develop mild spontaneous distal intestinal inflammation by the time they are 6 weeks old [14]. Colitic signs and symptoms become progressively worse chronologically. Colitis is therefore significantly worse in 15-week *Winnie* mice, when compared to 12-week *Winnie* mice. Corresponding commensurate histopathological findings have already been published [14].

**Figure 1:**
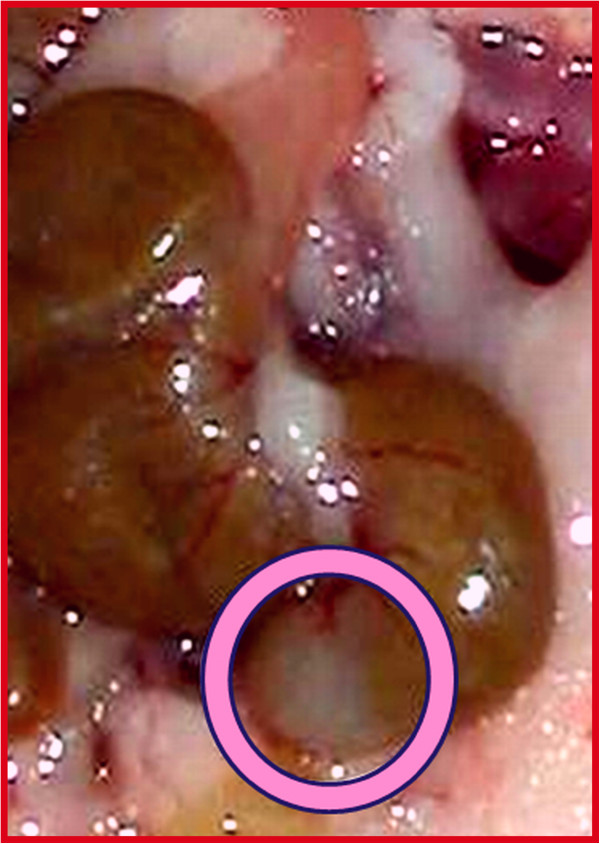
**The mouse caecal lymphoid patch (appendix).** Normal murine caecal lymphoid patch (≈human appendix) – This is the pale milky-white area encircled with a ring. [License number to reproduce image from John Wiley and Sons – 3415650102997].

A succinct summary and critical appraisal of more than a dozen studies by Koutroubakis et al. [15], show that appendicitis and appendectomy (AA) prevents or significantly ameliorates ulcerative colitis. In the mouse, the caecal lymphoid patch (Figure 1) is the rough equivalent of the human appendix. In Figure 1, this is the pale milky-white area encircled with a ring. AA *in the most proximal colon* substantially curbs T helper 17 cell -recruitment, −differentiation, −activation, and –effector (interleukin) expression *in the most distal colon*; thereby contributing significantly to suppressing Th17 pathway-mediated immunopathology in TNBS-colitis [16]. AA curbs autophagy [8], potentially contributing to suppression of autophagy-mediated immunopathology in colitis.

The appendix cannot be considered a “vestigial” organ “anymore”. Mouse models of spontaneous colitis have shown a significant role for the appendix in the adaptive immune response [17]. Appendectomy in these models also markedly reduced the risk for colitis development [17]. Several hypotheses exist explain this relationship, and, as more evidence endorses its important role in mucosal immunity [18]. The 2 most plausible explanations are as follows. The appendix may initiate an aberrant immune response against the gut microbiome, predisposing to IBD [19]. Alternatively, appendicitis and appendectomy may induce microbiota changes and/ or major immunological changes in the distal colon which protect against colitis development [8,16].

Most intestinal flora is anaerobic, and difficult to identify using culture-dependent methods [20]. However, next generation sequencing methods have enabled the high-throughput phylogenetic study of microbial populations from multiple samples in parallel [21]. The bacterial genome is assembled by amplification of fragments of the conserved 16S rRNA gene [22]. Hyper-variable regions within 16S rRNA are utilised to characterize different taxonomic levels [23]. In this study, we sought to investigate the microbiome signatures of *Winnie* mice in different stages of colitis development (at ages 12 and 15 weeks) and compare those against the control strain (C57BL/6). Microbiome alterations are expected between mice strains, as well as before and after inflammation. The rationale of our study was not only to determine what changes exist between a “normal” mouse strain, and a “spontaneously colitic” (*Winnie*) strain; but also to determine what bacteria were present at each stage of inflammation progression. These experiments will shed more light on the pathogenetic nuances of IBD. We compared stool and appendix phyla, as well as more intricate differences at the family level. Herewith, we posit the differences between the appendix and colon (stool), in normal controls and colitic *Winnie* mice.

## Results and discussion

### Results

#### Microbiome signature in winnie and C57BL/6

**Figure 2:**
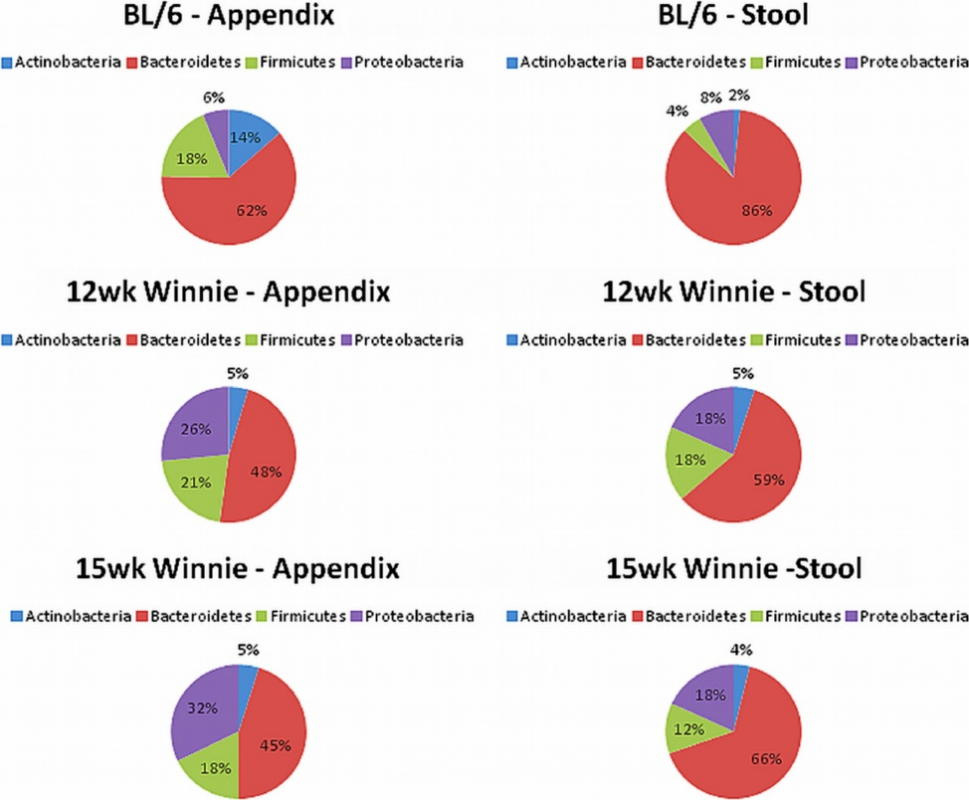
**Overview of most common bacterial colonies in mouse appendices and stool.** The *Bacteriodetes* phylum is the most abundant phylum in appendix and stool samples, from both C57BL/6 and *Winnie* mice. The *Proteobacteria* phylum is second most abundant in stool samples from both C57BL/6 and *Winnie* mice. The number of samples varied from 2–5 for each group.

**Figure 3:**
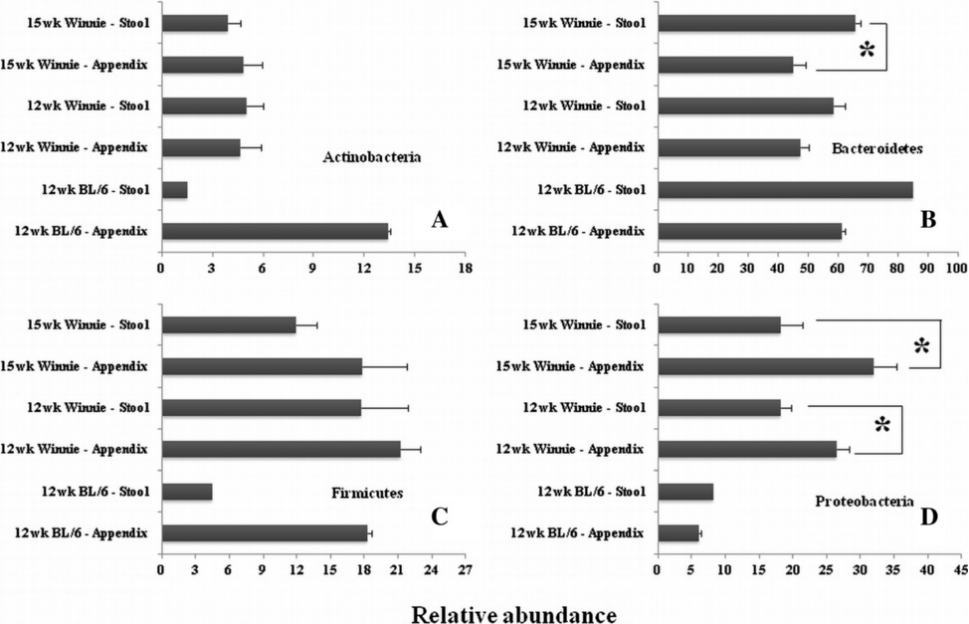
**Bacterial phyla signature of mouse appendix and stool specimens.** Bacterial phyla estimates in appendix and stool samples from *Winnie* and C57BL/6 mice aged 12 or 15 weeks were assessed: **(A)** Relative abundance estimate of phylum *Actinobacteria*. **(B)** Relative abundance estimate of phylum *Bacteroidetes*. **(C)** Relative abundance estimate of phylum *Firmicutes*. **(D)** Relative abundance estimate of phylum *Proteobacteria. Bacteriodetes* was significantly lesser in 15-week *Winnie* appendices compared to 15-week *Winnie* stool samples (P < 0.01). *Proteobacteria* was significantly increased in *Winnie* mice appendices (12 weeks) in contrast to stool samples (P < 0.05). *Proteobacteria* was increased in *Winnie* group appendices (15 weeks) compared to stool samples (P < 0.05). No microbiome signature differences were observed between *Winnie* stool samples, and between Winnie appendix samples. Error bars represent standard error of the mean. The number of samples varied from 2–5 for each group.

The *Bacteriodetes* phylum is the most abundant phylum in appendix and stool samples from both C57BL/6 and *Winnie* mice at both time-points (Figure 2). *Bacteriodetes* was significantly decreased (P < 0.01) in 15-week *Winnie* appendix samples compared to 15-week *Winnie* stool samples (Figure 3B). The *Proteobacteria* phylum was significantly more (P < 0.05) in appendix samples belonging to both time-points in *Winnie* mice (12 weeks and 15 weeks), when each was compared to stool samples (Figure 3D). No significant differences were observed between the microbiome signatures of *Winnie* stool samples. No significant differences were observed between the microbiome signatures of Winnie appendix samples.

#### Family-level bacteriodetes signatures in appendices

**Figure 4:**
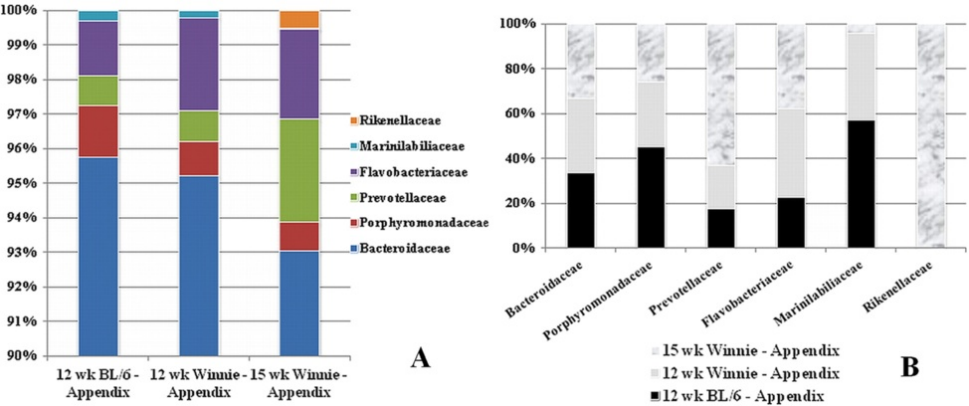
**Constituents and proportion of *Bacteroidetes *families in appendices. (A)** Relative proportions of different families in *Bacteroidetes* appendix samples from C57BL/6; and 12-week-, and 15-week *Winnie* mice. There are no statistically significant differences. **(B)** Individual *Bacteroidetes* family preponderances appendix samples from C57BL/6; and 12-week-, and 15-week *Winnie* mice. *Rikenellaceae* are found only in 15-week-old *Winnie* mice. C57BL6: 2 samples; 12-week Winnie: 4 samples; 15-week Winnie: 5 samples.

Family analyses of appendiceal *Bacteriodetes* phylum reveal no significant differences in family breakdown. *Bacteroidaceae* is the dominant (>90%) family in the appendices of both mouse strains, at both time-points (Figure 4A). There are no statistically significant differences in the constituents and proportion of *Bacteroidetes* families in appendices (Figure 4A). However, the *Bacteroidetes* family *Rikenellaceae* could be identified only in 15-week-old *Winnie* mice appendices (Figure 4B).

#### Family-level proteobacteria signatures in appendices

**Figure 5:**
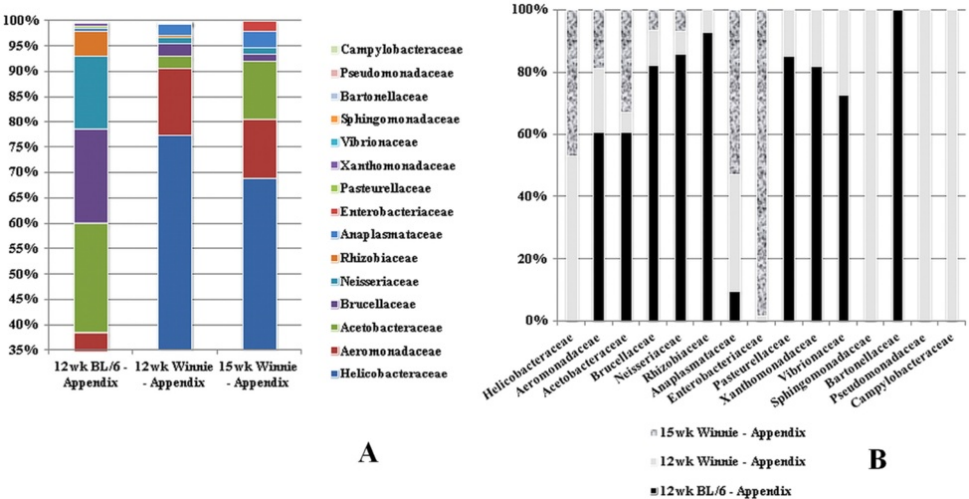
**Constituents and proportion of *Proteobacteria *families in appendices. (A)** Relative proportions of different families in *Proteobacteria* appendix samples from C57BL/6; and 12-week-, and 15-week *Winnie* mice. *Acetobacteraceae* are increased in 15-week Winnie mice when compared to 12-week Winnie mice (P < 0.05). **(B)** Individual *Proteobacteria* family preponderances appendix samples from C57BL/6; and 12-week-, and 15-week *Winnie* mice. *Helicobacteraceae* was not present in C57BL/6 controls. *Rhizobiaceae*, *Pasteurellaceae*, *Xanthomonadaceae*, *Vibrionaceae*, *Sphingomonadaceae*, *Bartonellaceae*, *Pseudomonadaceae*, and *Campylobacteraceae* which had trace presence in 12-week *Winnie* mice appendices, flatten out at the 15-week time-point. C57BL6: 2 samples; 12-week Winnie: 4 samples; 15-week Winnie: 5 samples.

Family analyses of appendiceal *Proteobacteria* phylum reveal significant *Acetobacteraceae* increases (P < 0.05) in 15-week *Winnie* mice when compared to 12-week Winnie mice (Figure 5A). *Helicobacteraceae*, which is prominent in *Winnie* mice at both time-points, are not represented in C57BL/6 (Figure 5B). The *Proteobacteria* families *Rhizobiaceae*, *Pasteurellaceae*, *Xanthomonadaceae*, *Vibrionaceae*, *Sphingomonadaceae*, *Bartonellaceae*, *Pseudomonadaceae*, and *Campylobacteraceae* which were present in traces in 12-week *Winnie* mice appendices, disappear at the 15-week time-point (Figure 5B).

## Discussion

The human vermiform appendix is commonly, but wrongly perceived to be a vestigial organ, despite its abundant lymphoid tissue, and despite the fact that appendicitis is the most common abdominal surgery requiring hospitalization. Anatomically, owing to its proximity to the caecal segment of the large intestine, it is perpetually exposed to and gastrointestinal microbiota. As the murine caecal lymphoid patch (Figure 1) is the equivalent of human appendix, this study investigated the microbial content differences of the caecal patch to the rest of colon (via stool contents).

**Figure 6:**
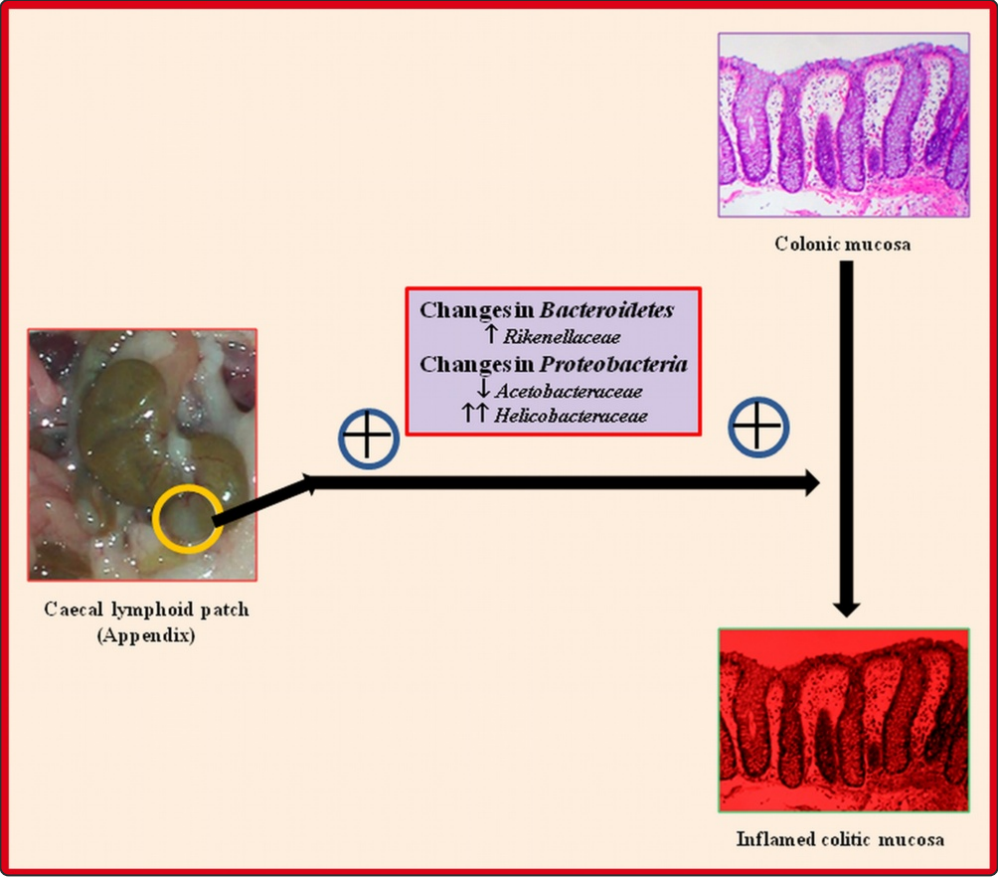
**The role of the appendiceal microbiome in experimental spontaneous colitis.** In this study, we note significant differences in appendiceal Proteobacterial compostion between controls and colitic (*Winnie*) mice. *Acetobacteraceae* is decreased in colitic mice. *Helicobacteraceae*, an unusual inhabitant of control mice appendices, is found abundantly in colitic mice. *Rikenellaceae*, a family belonging to the phylum *Bacterioidetes*, is found only in 15-week-old *Winnie* mice, but neither earlier, nor in controls.

*Bacteriodetes* was decreased in 15-week *Winnie* mice appendices compared to corresponding stool samples (Figure 3B). *Proteobacteria* increased in appendices of *Winnie* mice (12 weeks and 15 weeks), when each was compared to corresponding stool samples (Figure 3D). The *Bacteroidetes* family *Rikenellaceae* could be identified only in 15-week-old *Winnie* mice appendices (Figure 4B, Figure 6). Significantly more *Acetobacteraceae* (*Proteobacteria* phylum) was present in 15-week *Winnie* mice when compared to 12-week Winnie mice (Figure 5A, Figure 6). *Helicobacteraceae* (*Proteobacteria* phylum), prominent in all *Winnie* mice, is absent in C57BL/6 control mice (Figure 5B, Figure 6).

Intestinal biopsies from IBD patients display marked aberrations in the microbiome signature [24], inclusive of its *Proteobacteria* and *Bacteriodetes* constituents [25]. Surgical samples from IBD patients show reduced *Bacteroidetes* load compared to healthy patients [26]. Colonic biopsies show significantly more *Proteobacteria* in IBD patients [27]. *Bacteroidetes* species are an important source of short chain fatty acids to the intestinal epithelia [28]. In addition, *Bacteroidetes* regulate the generation of colonic regulatory T cells, which maintain immunological tolerance against the gut microbiome [29]. Specifically, capsular polysaccharide A from *Bacteroides fragilis* stimulates colonic regulatory T cells enhances the release of the anti-inflammatory cytokine IL-10 [30].

*Proteobacteria* levels are higher in IBD patients [31]. Multiple classes of *Proteobacteria* are associated with IBD including *Deltaproteobacteria*, which include sulphur-reducing bacteria [32]. Increased sulphide damages intestinal walls [33] and inhibits butyrate oxidation [34]. More importantly, *Helicobacter* species, which was represented in *Winnie* mice but not in C57BL/6 (Figure 5B, Figure 6), is strongly associated with clinical IBD (UC > CD) [35]. The increase in *Helicobacteraceae* in IBD patients is not due to *Helicobacter pylori* infection, as *Helicobacteraceae* is prominent in colonic but not gastric mucosa [36].

Limitations of our study include small sample sizes, and methodological differences (with other studies) in DNA extraction/sequencing, which may hypothetically impart an element of inconsistency. Firstly, the QIAamp DNA Stool Mini kit we used relies on enzymatic lysis, and produces substantially less DNA yield, compared to extraction methods involving vigorous mechanical lysis [37], or bead-beating [38]. This may have a disproportionate impact on specific bacterial groups which are tougher to lyse owing to stronger cell walls [39]. In addition, pyrosequencing can be biased by selection of the primer pair, and amplifying different 16S rRNA hyper-variable regions may enhance sequencing coverage [40].

Our study suggests an association or a pathogenic role for the appendix and its flora in colitic predisposition. Further characterisation of chronological differences in inflammation progression, as well as the magnitude of colitis pathology; would divulge whether our observed microbiome changes indicate association or causality. Dysbiosis within the appendix (caecal patch) of our colitis model was significantly more than within stool samples, especially in older (15-week) *Winnie* mice, where intestinal epithelial defects become pronounced [41]. It is not clear whether dysbiosis is a cause or consequence of intestinal inflammation [42]. However, it is less likely to be the former, owing to the therapeutic benefit easily conferred by gut microbiome manipulation [10]. Reversing the dysbiotic-inflammatory positive feedback loop with faecal microbiota transplantation has produced positive results in recurrent *Clostridium difficile*-associated disease (CDAD) [43]. While the appendix might promote *C. difficile* infection [44], retrospective studies in humans show a protective role against CDAD recurrence [45]. Additional research in experimental models is needed to explain these discrepancies.

## Conclusions

The correlation between dysbiosis and IBD is incompletely understood. Our *Winnie* mouse strain has an altered intestinal barrier, resulting in spontaneous colitis, characterized by intestinal ulceration and inflammation. The appendiceal dysbiosis observed in our colitic *Winnie* mice is commensurate with, and adds to extant literature data. The presence of *Helicobacteraceae* (*Proteobacteria*) only in colitic *Winnie* mice (but not control mice) is consistent with reports of increased *Helicobacter* in IBD patients. *Bacteroides* (*Bacteroidetes*) decreases may be a reflection of reduced anti-inflammatory commensal species such as *B. fragilis*. Further research is warranted to expand and delineate the relationship between IBD and the appendix microbiome. Despite inherent differences between mouse and human gut flora, this study using our *Winnie* colitis model will vastly increase our understanding of IBD pathogenesis.

## Methods

### Mice

Two C57BL/6 and 18 *Winnie* mice were purchased from the Animal Resource Centre, Australia. Owing to discrepancies in the supply of mice strains, there were mild differences in the number of animals in each experimental group. The mice were bred in a conventional clean *Helicobacter hepaticus* - free animal facility used for all the experiments, which were conducted under the directions and approval of the animal ethics committee of the University of Queensland.

### Caecal patch (appendix) excision and DNA extraction

Caecal patch was identified as a whitish-patch segment right at the end portion away from the colonic side (Figure 1). About 1 cm excision was made and the sample with contents was immediately snap-frozen for further DNA processing. In the laboratory, stool material was scraped and DNA extracted using QIAamp DNA mini kit (Qiagen, Hilden, Germany). Bacterial DNA content was confirmed with bacterial 16 s rRNA broad-range primers.

### DNA extraction

Mice were euthanized at ages 12 and 15 weeks and appendix and stool samples were immediately frozen and stored at −80°C. DNA was extracted using the QIAamp DNA Stool Mini Kit (Qiagen, Hilden, GER), according to manufacturer’s instructions then stored in −20°C. Bacterial DNA content was confirmed using the A260/A280 ratio and real-time PCR of 16S rRNA using broad-range primers as described previously [46] on the iQ5 real-time PCR system (Bio-RAD Laboratories, CA, USA).

### DNA sequencing and metagenomic analysis

Sequencing amplicons were generated for the V1-V3 hypervariable region of the 16S rRNA gene using the 27 F-519R primers and DNA was sequenced using the Roche GS FLX + 454-pyrosequencing platform at the Australian Genome Research Facility. Profiling of the microbiome was performed using the metagenomic tools available in the Galaxy Public Server [47] following the metagenomic pipeline described by Kasakovsky Pond et al. [48]. Briefly, sequences were filtered to exclude reads with a quality score <20 and a contiguous length <250 bp. Sequences were compared to the WGS database (28jan2013) using MegaBLAST z [49] and after removing hits with <50% coverage, Operational Taxonomic Units (OTU) were designated based on 80% similarity and identity defined to the lowest taxonomic rank of kingdom.

### Statistical analysis

Since our results could not form a normal distribution due to the small sample size, we used the non-parametric Mann–Whitney U-test. Graphs were generated with Prism v6 (GraphPad Software, CA, USA).

## Abbreviations

IBD: Inflammatory bowel disease; CD: Crohn’s disease; UC: Ulcerative colitis; NOD2: Nucleotide-binding oligomerization domain-containing protein 2; ATG16L1: Autophagy related 16-like 1; IL-23: Interleukin-23; Th17: T helper 17; AA: Appendicitis and appendectomy; 16S rRNA: 16S ribosomal RNA; TNBS: 2,4,6-Trinitrobenzenesulfonic acid; OTU: Operational taxonomic unit.

## Competing interest

The authors declare that they have no competing interests.

## Author contributions

RE and DK - Conception and design; SA, DK, SR-D - Research work; SA, RC, DK, SR-D, RE – Figure preparation & Manuscript writing. All authors read and approved the final manuscript.
